# Impact of the dog population and household environment for the maintenance of natural foci of *Leishmania infantum* transmission to human and animal hosts in endemic areas for visceral leishmaniasis in Sao Paulo state, Brazil

**DOI:** 10.1371/journal.pone.0256534

**Published:** 2021-08-31

**Authors:** Patricia Sayuri Silvestre Matsumoto, Roberto Mitsuyoshi Hiramoto, Virgínia Bodelão Richini Pereira, Valéria Medina Camprigher, Helena Hilomi Taniguchi, José Eduardo de Raeffray Barbosa, Luiz Ricardo Paes de Barros Cortez, Elivelton da Silva Fonseca, Raul Borges Guimarães, José Eduardo Tolezano

**Affiliations:** 1 Parasitology and Mycology Center, Adolfo Lutz Institute (IAL), Sao Paulo, Sao Paulo, Brazil; 2 Adolfo Lutz Institute, Regional Laboratories Center II Bauru, Bauru, Sao Paulo, Brazil; 3 Center for Zoonoses Control of Bauru, Health Secretariat of Bauru, Bauru, Brazil; 4 Bioterium nucleos, Adolfo Lutz Institute (IAL), Sao Paulo, Sao Paulo, Brazil; 5 Institute of Geography, Federal University of Uberlândia, Uberlândia, Minas Gerais, Brazil; 6 Department of Geography, Sao Paulo State University/Faculty of Sciences and Technology (FCT/UNESP), Presidente Prudente, Sao Paulo, Brazil; Universidade Federal da Bahia, BRAZIL

## Abstract

When it comes to visceral leishmaniasis (VL) in Brazil, one of the main targets of public health policies of surveillance is the control of domestic canine reservoirs of *Leishmania infantum*. This paper aims to evaluate the effect of the dog population and household environment for the maintenance of natural foci in the transmission to human and animal hosts in an endemic city for VL, Bauru, in Brazil. We collected 6,578 blood samples of dogs living in 3,916 households from Nov.2019 to Mar.2020 and applied geospatial models to predict the disease risk based on the canine population. We used Kernel density estimation, cluster analysis, geostatistics, and Generalized Additive Models (GAM). To validate our models, we used cross-validation and created a receiver operating characteristic (ROC) curve. We found an overall canine VL (CVL) seroprevalence of 5.6% for the sampled dogs, while for the households, the positivity rate was 8.7%. Odds ratios (OR) for CVL increased progressively according to the number of canines for >2 dogs (OR 2.70); households that already had CVL in the past increased the chances for CVL currently (OR 2.73); and the cases of CVL increase the chances for human VL cases (OR 1.16). Our models were statistically significant and demonstrated a spatial association between canine and human disease cases, mainly in VL foci that remain endemic. Although the Kernel density ratio map had the best performance (AUC = 82), all the models showed high risk in the city’s northwest area. Canine population dynamics must be considered in public policies, and geospatial methods may help target priority areas and planning VL surveillance in low and middle-income countries.

## 1. Introduction

Leishmaniasis is a group of infectious diseases caused by a protozoan of the *Leishmania* genus that affects humans and animals. The transmission occurs by the bite of the dipterous of the subfamily Phlebotominae, the sand flies. It is considered one of the most widely distributed neglected diseases worldwide [1], being a health problem in North and East Africa, West and East Asia, and the Americas [2].

Leishmaniasis is a focal disease, and its epidemiology differs according to nosogeography entity, which means that different spatial patterns for each species of *Leishmania* occur, and different strategies for the control of leishmaniasis are demanded. Visceral leishmaniasis (VL), for instance, is one of the most severe leishmaniasis. It threatens more than one billion and a half persons living in at-risk areas around the world. In 2015, between 50 and 90 thousand new cases were estimated per year, with an incidence rate of 2.27 per 100 thousand inhabitants. Only six countries, including Brazil, accounted for about 90% of all new cases [3, 4]. In the last update in 2021, Brazil notified 51,931 cases from 2005 to 2019, a mean of 3,462 per year [2].

The main reservoir of *L*. *infantum* in domestic habitats is the domestic dog [5, 6]. However, for control programs, an integrated knowledge about the ecological niche of the vector and environmental conditions is fundamental to address effective measures [7]. For this reason, geospatial modeling is a valuable instrument to target interventions of control programs [8].

In Brazil, there is a great difficulty for the effective implementation and operation of the VL control programs [9]. Overall, the Brazilian Visceral Leishmaniasis Control Program (VLCP) is based on canine reservoirs’ control, consisting of serosurvey and culling of dogs; control of the vector spraying insecticides inside the households and environmental management; and early diagnosis and treatment of human cases [8].

The first evidence of VL in Sao Paulo state was the discovery of *Lutzomyia longipalpis* in the urban area of Araçatuba municipality in 1997 [10]. In 1998, autochthonous canine cases of VL were reported, and for the first time in the state, the sand fly was suspected as the vector. In 1999 autochthonous human cases were reported for the first time [11, 12].

Regarding public health, several factors may be responsible for the persistence of the number of cases and deaths in Sao Paulo state, such as difficulty for early diagnosis and specific treatment in humans; difficulty in the correct identification and control of domestic reservoirs; and difficulty in controlling vector population [6, 13]. In addition, there is unclear knowledge about other determinants that may influence the design of novel strategies for the control and prevention of VL [14].

In Sao Paulo state, the municipality of Bauru had the first evidence of VL in 2002, when the sandflies were found, and the first autochthonous infection in a dog was reported. The first human records occurred in 2003. Since that time, there have been 580 cases and 46 deaths, a lethality rate of 7.9% from 2003 to 2020 [15]. Bauru was chosen to perform this research because of its high number of cases and endemicity in the region. In Bauru, there is a lack of information about the spatial distribution and a long-term follow-up of CVL, which could aid in the global understanding of the problem. Mapping the exact occurrence of the human or canine cases may help better understand the disease and plan public policies regarding VL.

This study aimed to calculate the impact of the household environment and canine population for visceral leishmaniasis risk through geospatial methods. We hypothesize that: a) in endemic areas for VL, a higher number of dogs in the households increases human or canine VL cases. b) the urban area is stratified by different geographical profiles that allow the remaining endemicity, needing targeted strategies as control measures. Using spatial analysis and statistical approaches, we constructed a space framework based on a large serosurvey conducted between November 2019 and March 2020 in the urban area of Bauru.

## 2. Materials and methods

**Fig 1:**
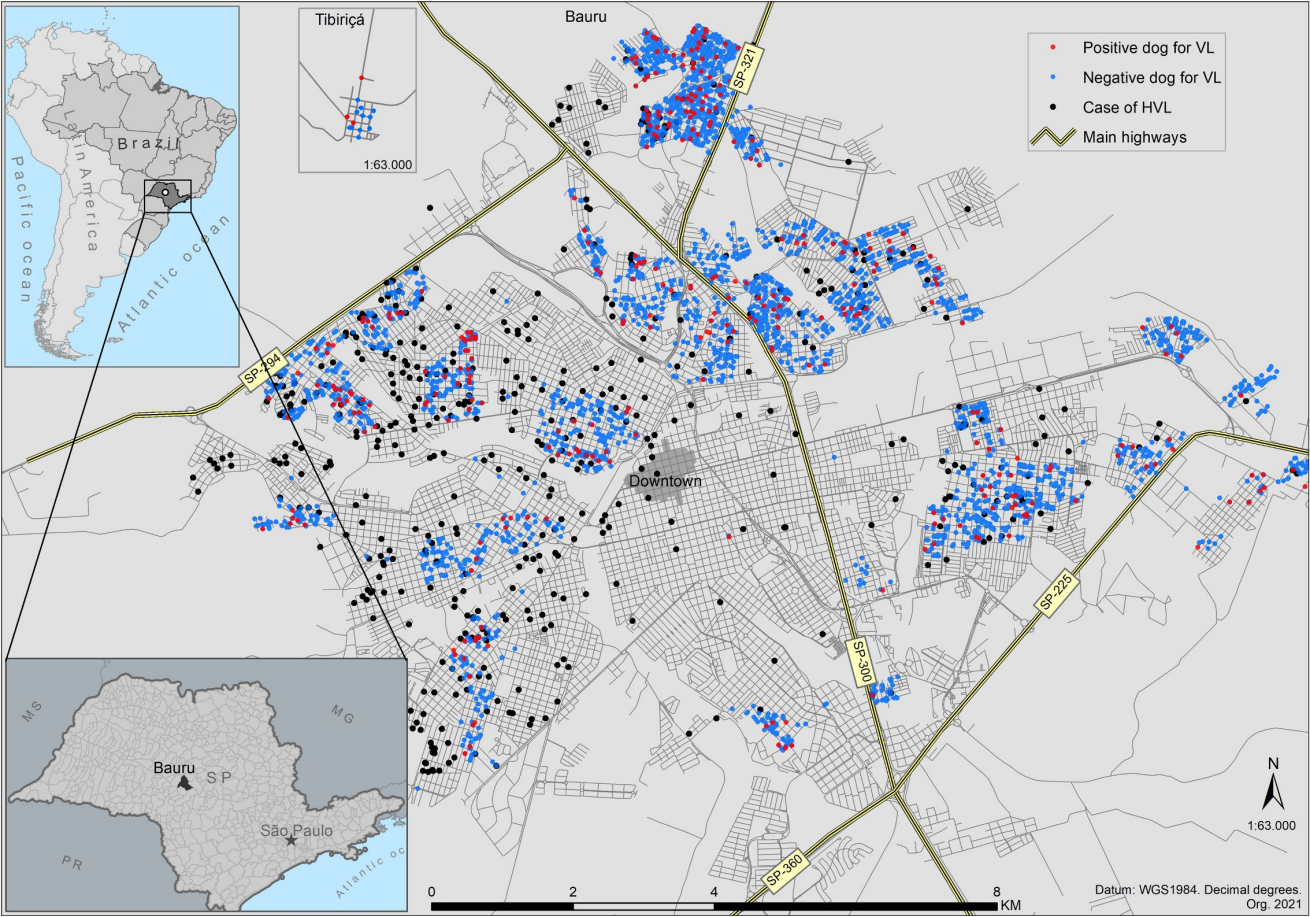
Geospatial location of human cases of VL and the serosurvey in Bauru’s urban area. A total of 6,578 blood samples of dogs were analyzed. Points represent each dog’s address. Positive dogs for CVL are represented by red dots and negative by blue. Black dots are HVL cases. Points are overlapped because of the spatial resolution of the cartographic scale.

Bauru is a central municipality in the state of Sao Paulo (22°18’52" S, 49°03’31" W). It is crossed by important highways: SP-300 Marechal Cândido Rondon Highway, SP-294 Comandante João Ribeiro de Barros Highway, SP-321 Cezário José de Castilho Highway, and SP-225 Engenheiro João Batista Cabral Rennó Highway, giving access to the countryside cities of the state and the capital Sao Paulo (Fig 1).

According to the Köppen-Geiger climate classification updated system [16, 17], Bauru climate is classified as Cfa, which means temperate, without dry season, and with hot Summer. The soil is unsaturated, reddish and dark brown, fine clay sand texture, underlain by sandstone of the Bauru group. The vegetation of the urban area is Tropical Semideciduous Forest mixed with Cerrado, highly impacted by urbanization variable patchy pattern. The average altitude of Bauru is 527.4 m.

The population comprises an estimative of 379,297 inhabitants [18]. A research was conducted in 20,958 households in Sao Paulo state countryside, in which 52.6% proven dog ownership, with an average of 1.6 dogs at home and an inhabitant ratio of 1:4 dogs per person [19]. Following this study, we estimated the dog population at 99,815, according to the Brazilian Geographic and Statistics Institute [18].

### 2.1. Study design

**Fig 2:**
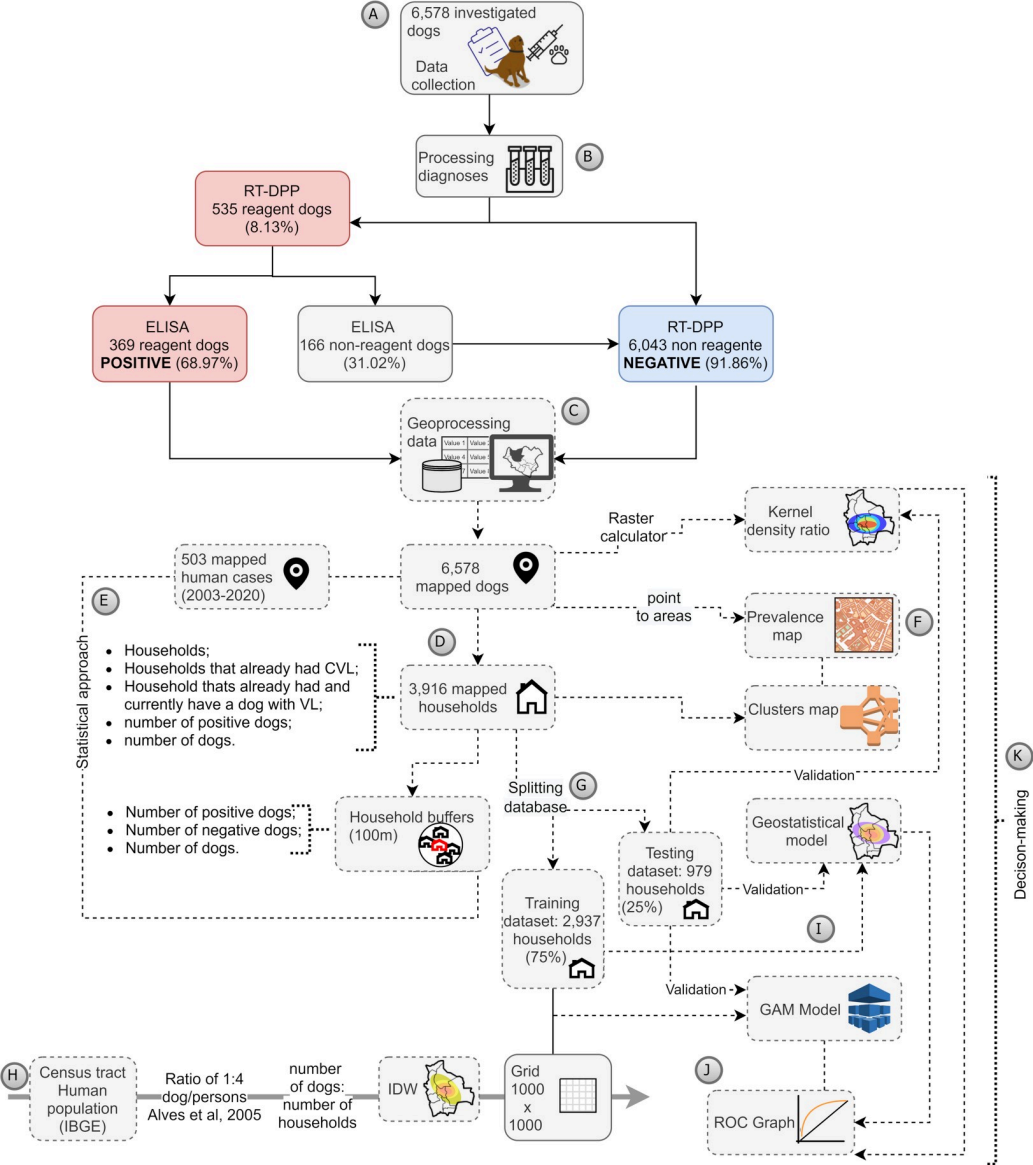
Synthesis of the performed methodology. The methodological proceedings were performed according to (a) Collecting samples and guardian’s survey. (b) Analyzing the samples. (c) Mapping the samples of dogs and human cases of VL. (d) Changing the scale of the dogs into households. (e) Applying statistical models. (f) Performing thematic maps. (g) Preparing data for the spatial model. (h) Preparing training data for the spatial model. (i) Validation of the models. (j) Using acquired knowledge for decision-making.

The canine serosurvey was conducted from December 2019 to March 2020. Agents of the Center for Zoonoses Control visited 3,916 households to collect the blood samples of 6,578 canines. In addition, a short survey was applied (S1 Fig) to the dog’s guardian to identify the previous presence of an infected dog in the household (Fig 2A). We tested and mapped the samples (Fig 2B and 2C), and then we used spatial analysis to prepare data for creating thematic maps (Fig 2F) and statistical models (Fig 2D–2I).

We applied a framework of starting with a statistical to select variables most influential in running the spatial models. The chosen method of statistics was the binary logistic regression, used to associate the size of the canine population related to the odds ratio of having VL cases. As the results of the statistical model were statistically significant, we ran the spatial models. The generalized additive model—GAM, geostatistic model, and Kernel density ratio were chosen to identify the spatial dependence of the cases and their spatial association with the number of the canine population, emphasizing hotspots of CVL. We finally validated our model using cross-validation (Fig 2F and 2J). The methodology performed here will be detailed in the following sections.

#### 2.1.1 Definition of cases

Dual-Path Platform rapid test (TR-DPP, Biomanguinhos®, Rio de Janeiro, Brazil,) is used by the Brazilian VLCP to test the samples. The TR-DPP® is a test for *Leishmania infantum* based on the reaction of IgG to the antigen K28. Enzyme Linked Immunosorbent Assay ELISA—Biomanguinhos® is used to confirm the positive diagnoses. ELISA is characterized by the reaction of soluble and purified antigens of *Leishmania* promastigotes, obtained from cultures and adsorbed in microtiter wells with *Leishmania*-specific antibodies present in serum samples. The diagnostic was run in a Multiscan spectrophotometer using a 450 nm filter and cutoff values ("Cutoff" = CO): CO = average negative controls x 2. The diagnoses were performed according to the manufacturer’s instructions and the directions of the VLCP.

A combination of TR DPP® and ELISA reagent was considered a positive result according to the Brazilian VLCP recommendations for canine diagnoses, routinely used by the Centers for Zoonoses Control in Sao Paulo [20]. TR DPP® non-reagent was considered a negative result–Fig 2A. The prevalence was calculated based on the outcome, being a proportion of a dog found positive for CVL divided by the sampled dog population. The guardian of the dog provided consent for sample collection involving domestic dogs in the areas surveyed. All serosurvey was supervised by the veterinary group of the Center for Zoonoses Control in Bauru municipality. Households without dogs, closed or that refused to give consent were excluded from the analysis.

The human laboratory diagnoses are based mainly on serological methods and microscopic diagnoses (parasitological). When amastigotes are identified, it is considered a certainty diagnostic. Patients with clinical manifestation and reagent rapid immunochromatographic test rK39 and/or Indirect immunofluorescence with titers equal to or greater than 80 are considered positive for VL [20]. Human cases addresses come from the epidemiological surveillance center (CVE), State Health Secretariat [15] and comprehend secondary databases from the Brazilian System for Notifiable Diseases (SINAN) in the period from 2003 to 2020.

This study was approved under number 03/2000 in the Ethics Committee on the Use of Animals in Research at the Adolfo Lutz Institute, Sao Paulo-SP, Brazil.

#### 2.1.2. Mapping

The addresses of dogs and the human cases were geocoded by an application programming interface (API) of Google Maps (Google®), based on the municipality’s cartographic street map. A lower score of geocoding was topologically adjusted to ensure the correction of georeferencing. Point features were plotted in a Geographic Information Systems (GIS) ArcGIS 10.2.2 (ESRI, Imagem).

**Fig 3:**
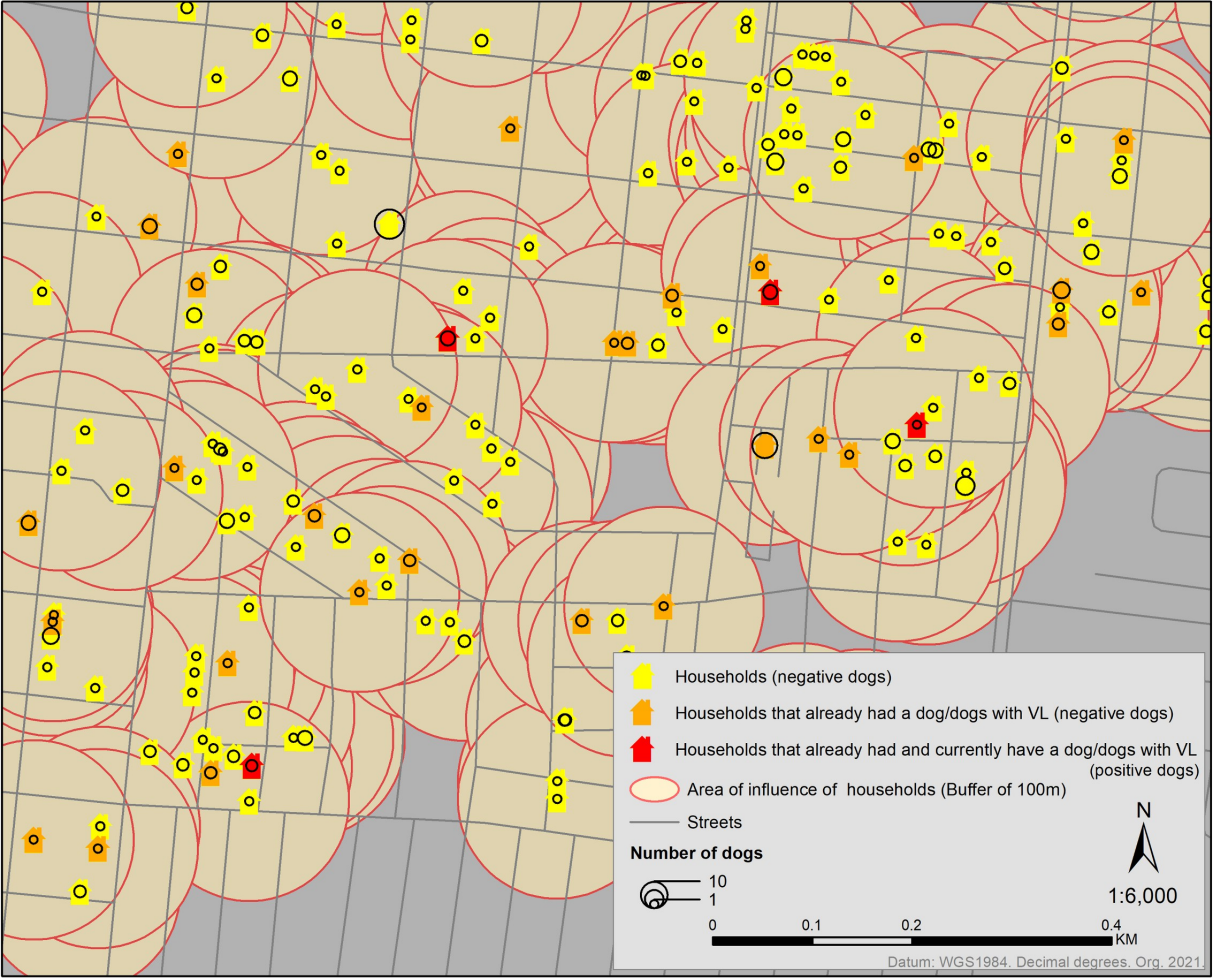
Geocoded households of Bauru, stratified by dog population, positive dogs, and buffer zones. The households are identified according to the conducted surveys. Yellow, orange, or red symbols represent the dog’s households sampled. Yellow had not an infected dog; orange had an infected dog (in the past); red had and currently has an infected dog. Proportional circles represent the number of dogs in each household. We created a buffer of 100m in each sampled household to calculate the number of dogs, positive or negative dogs in this area.

Canine data and the households were mapped as point data; however, they have different cartographic scales once the household (mapped by address) is the boundary, besides dogs are also represented by geocoded addresses. It means that more than one dog in a household is visualized as one point, but there are overlapped(s) dog(s) (points) in there. Bearing this in mind, the dog layer was transformed into the household layer with the number of dogs (Fig 2C and 2D) and categorized as negative or positive for VL in each survey. Fig 3 shows the mapped data.

To run the Generalized Additive Model (GAM), we created a fishnet grid of 1000x1000 of 50m containing the estimated number of dogs per domicile in each coordinated. We calculated the number of dogs based on the human population by census tracts [21], in a ratio of 1:4 dogs/inhabitants [19]. The mean number of dogs was divided by the mean number of households in each census tract. We then created a centroid and calculated the Inverse distance weighted (IDW) interpolation, considering the mean number of dogs per domicile. The grid values were extracted from the raster surface generated by the IDW (S7 and S8 Figs).

To analyze the area of influence of households with infected dogs in the environment, we created buffer zones of 100m (Fig 3). We then calculated the number of dogs, negative dogs, and positive dogs using spatial analysis tools. Finally, we aggregated features of point data into polygons, using the census tracts database, to stratify the prevalence spatially.

### 2.2. Statistical and spatial analysis

For all the performed calculations, we considered a significant value at p≤0.05. We used the geographic information system (GIS) ArcGIS 10.2.2 and R language, with several packages described in the sections below.

The number of dogs per household was categorized as binary to verify each group, for instance, households that have only one dog (1 = 1 dog and 0 > 2 dogs); two dogs (1 = 2 dogs; 0 = 1 or > 2 dogs); and two or more dogs (1 = > 2 and 0 = 1 or 2 dogs). Households that already had a positive dog or a human case were also categorized as binary, e.g., 1 for cases and 0 for non-cases.

#### 2.2.1. Pearson’s correlation

We calculated Pearson’s correlation to identify a possible association between the number of cases of CVL and: i) the number of investigated samples or ii) the number of households that already had an infected dog, or iii) the number of households that already had and currently have an infected dog/dogs.

#### 2.2.2. Binary logistic regression

We tested if the households with an infected dog (outcome = 0 for a household with no infected dog/dogs or outcome = 1 for a household with an infected dog/dogs) or an area of influence of household (outcome = 0 for areas of influence of household with no infected dog/dogs or outcome = 1 for an area of influence of household with infected dog/dogs) could increase the chances to have cases of the disease. We used ’oddsratio’ package in RStudio (4.0.0).

#### 2.2.3. K-function

Being aware of spatial dependence of CVL promoting different risks or protection, we evaluate, locally, the spatial interactions in the urban neighborhoods. Ripley’s K-function with 999 permutations was applied to identify households’ spatial patterns at distances [22]. In this function, K(t) is the number of events within a distance of an arbitrary event, divided by the overall density of events. We plotted maximum and minimal envelopes of K(t) simulated values, giving the statistical significance for clustered or dispersed patterns (S2 Fig).

#### 2.2.4. Cluster analysis

We used cluster analysis to detect significant concentrations of CVL within Confidence Intervals (CI) of 90%, 95%, and 99%—S5 Fig. Clusters were calculated using the Getis-Ord Gi statistic, which identifies features with high or low values of a spatial cluster. The pattern can be expressed by clustered, dispersed, or random features representing a measurable spatial aggregation unit.

#### 2.2.5. Kernel density

Using the K-function dependence, we chose the minimal distance of concentration of our data, 0.5 km, to set the bandwidth. We used the quartic kernel function [23], which is given by

$$\hat{\lambda_{\tau }}\;(s)=\Sigma_{d_{i}\leq \tau }\frac{3}{\pi \tau^{2}}\;{\left(1-\frac{d_{i}^{2}}{\tau^{2}}\right)}^{2}$$ (1)

where:

i = *1*,*…*,*n* are the input points.
d_i_ is the distance between the point s and the observed event in location,
s_i_ and τ is the radius centered on s.

We plotted Kernel density maps for CVL cases (S3 Fig) and canine samples (S4 Fig). A Kernel density ratio map was then performed (CLV: samples), which gives a visualization of the risk for the disease.

#### 2.2.6. Geostatistical approach

According to the number of dogs, a geostatistical approach was performed to predict the higher risk areas for CVL. We used the Ordinary Kriging method and select two datasets: cases of CVL and number of dogs. We adjusted data in a stable model in a semivariogram, in which for a set of experimental values z(x) and Z (x1+*h*), separated by *h* distance, is defined by the Eq 2:

$$\sigma (h)=\frac{1}{2N(h)}\sum_{i=1}^{N(h)}{\left[z\;(x_{i})-z(x_{i}+h)\right]}^{2}$$ (2)

Where,

*N(h)* is the number of experimental pairs;
*h* is the regular interval that separates *z(xi)* e *z(x*_*i*_*+h)*

Geostatistics parameters were adjusted as follow: number maximum and minimal neighbours = 5 and 2, respectively; lags = 12; lag size = 0.64; nugget = 0.46; range = 3.8974; sill = 0.062 and 45 degrees–S6 Fig and S1 File.

#### 2.2.7. Generalized additive model

We run a GAM according to an approach reported for case-control data [24, 25], in which we considered *Y*_*i*_ = 1 (cases) and *Y*_*i*_ = 0 (non-cases), *d* is the number of dogs in location *i*, and P(*Y*_*i*_ = 1|*d*_*i*_ *S*_*i*_) is calculated according to Eq 3:

$$\text{logit}({\text{P}}_{\text{i}})=\text{a}+\text{b}\times {\text{d}}_{\text{i}}+{\text{S}}_{\text{i}}$$ (3)

where,

a is the ratio of cases to non-cases,
b is the coefficient for the number of dogs per household,
S_i_ is a function of the residual spatial variation after accounting for the effect of the number of dogs.

We model S_i_ by a locally estimated scatterplot smoothing (LOESS) regression smoother against the Universal Transverse Mercator coordinates. We choose the optimal smoother parameter of the models based on Akaike’s Information Criterion (AIC) [24] after testing multiple bandwidths (S2 File). We predicted the adjusted log odds for each location and omitted the covariate and smoothing terms through a null model. GAM was run in RStudio using the ’gam’ package.

### 2.3. Cross-validation

For further analysis, we validated our models using cross-validation. We created random samples in ArcGIS and split our database into training (75%, 2,937 points) and testing (25%, 979 points). Spatial models were created using the training dataset to predict the risk for the testing dataset. For each model, the best threshold was chosen, and we calculated specificity, sensitivity, and accuracy for correctly predicting the observed value of a case or non-case at the testing coordinates. To sum up, we calculated the area under the receiver operating characteristic (ROC) curve (AUC) with 95% confidence interval, which plots the true positive rate versus false positive rate, allowing identifying the performance of the models. We used the ’pROC’ and ’ggplot2’ packages in RStudio.

## 3. Results

**Fig 4:**
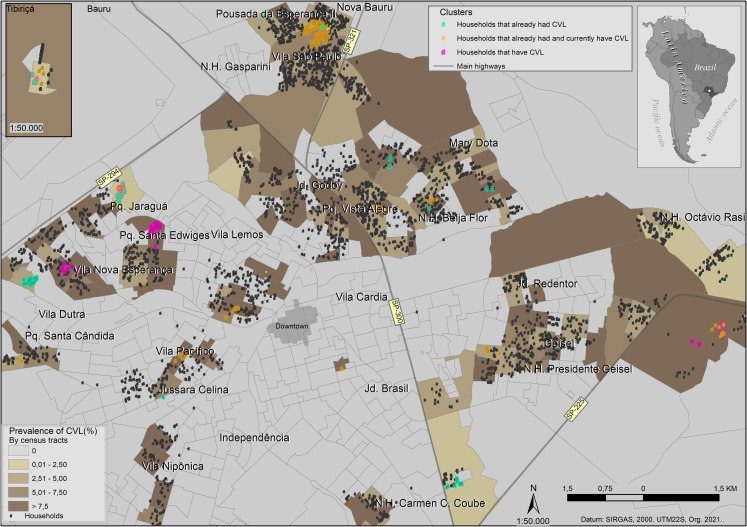
Geocoded households of Bauru, stratified by prevalence and cluster analysis. Each black dot is a household with no identified cluster. Magenta dots are the clusters of domiciles with infected dogs; green dots are the clusters of households that already had infected dogs; and orange dots represent the clusters of households that had and currently have infected dogs. Different size symbols and opacity households were set to ensure the spatial visualization of overlapped households.

We investigated 6,578 dogs, in which we found Anti-*Leishmania* spp. antibodies in 8.1% of TR DPP® (535/6,578) and 5.6% in both TR DPP® and ELISA (369/6,578). We found different spatial prevalence in the city, ranging from 0 to 50%. The mean prevalence was 2.67%, and the higher prevalence (>7.5%) was regularly distributed in the sampled area (Fig 4).

### 3.1. Cluster analysis

We identified a clustered pattern of households with CVL with statistical significance from approximately 0.5 to 6.5 km, and a clustered pattern of human cases from 0.5 to 4 km (S2 Fig). We found spatial clusters of high values (hot spots) in the west, north, east, south, northeast, southwest, southeast, and in Tibiriçá, a municipality district (Fig 4).

### 3.2. Kernel’s map

**Fig 5:**
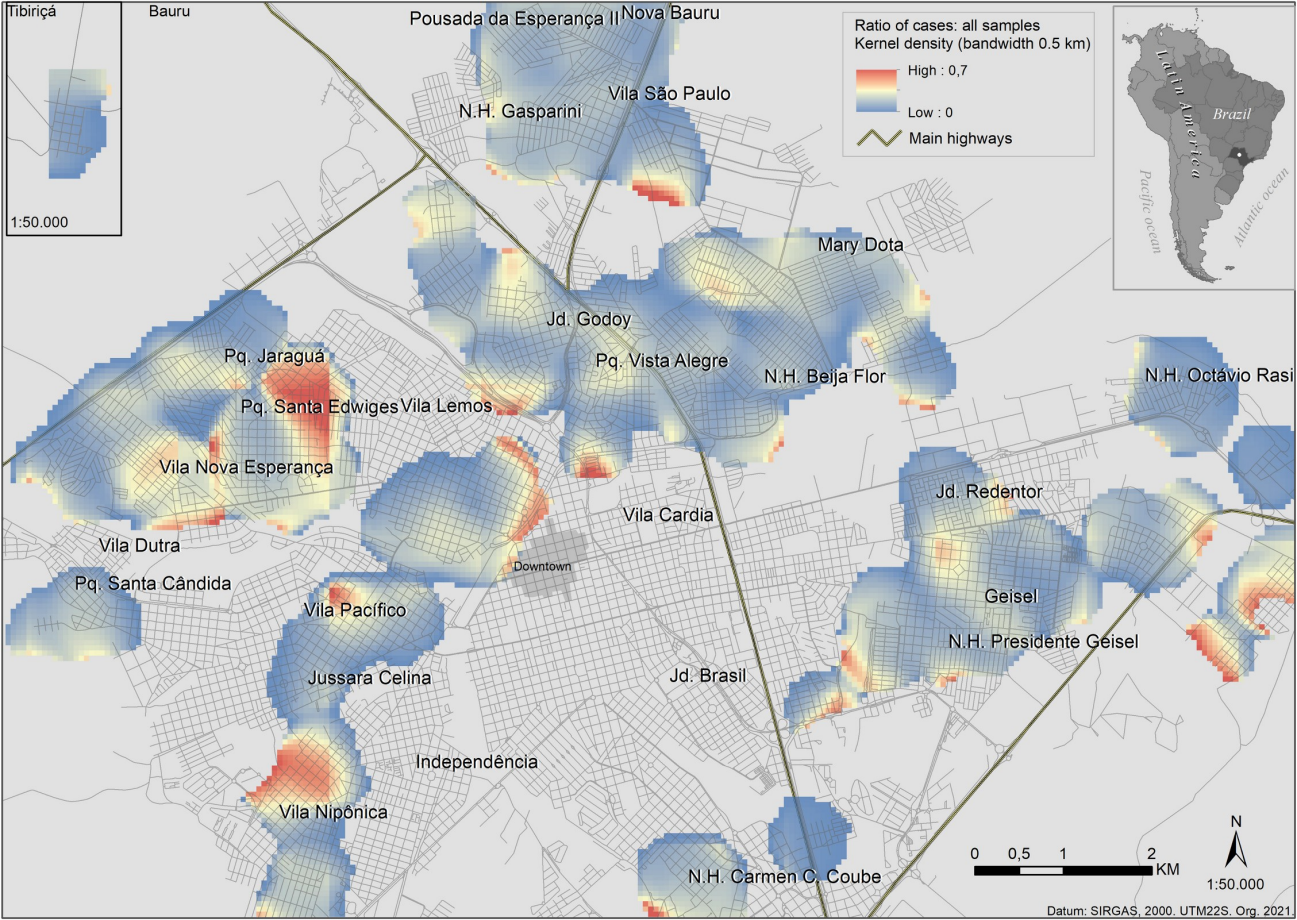
Kernel density ratio for canine visceral leishmaniasis. Kernel density ratio map ranging from 0 (blue) to 0.7 (red), which gives a visualization of the risk dividing the concentration of cases of CVL (S3 Fig) by the concentration of dog samples (S4 Fig). The areas of higher risk are in the west and southwest.

The ratio of cases per sample concentration represented in the Kernel map shows high-risk areas in the Pq. Jaraguá, Pq. Santa Edwiges and Vila Nipônica neighborhoods (Fig 5). Other high-risk concentrations in Kernel’s map represent the border effect.

### 3.3. Pearson correlation

Pearson’s correlation was positive and moderate considering the number of infected dogs and the households investigated (0.508, p-value = 0.000); positive low for infected dogs and the households that already had an infected dog/dogs (0.240, p-value = 0.000); and positive and low for infected dogs and the households that already had and currently have a dog/dogs with VL (0.129, p-value = 0.000). All conditions were statistically significant.

### 3.4. Binary logistic regression for visceral leishmaniasis

We investigated 3,916 households, in which 16.7% (656/3,916) already had a positive dog—independently when it was (Table 1). Nowadays, 8,7% (341/3,916) of the households have at least one positive dog. From the households with positive dog/dogs in the past, 17.0% (112/656) still have positive dog/dogs currently.

**Table 1:** CVL diagnostic, dog count in the households, and buffer zone extraction versus the number of sampled dogs in Bauru.

Category	Description	*n*	%
*Diagnoses*	Investigated dogs (samples)	6,578	100
	Reagents samples (TR DPP®)	535	8.1
	Non-reagents samples (TR DPP®)	6,040	91.8
	Positive dogs (reagent for TR DPP® and ELISA)	369	5.6
*Households*	Investigated households	3,916	100
	Households with only one dog	2,269	57.9
	Households with two dogs	1,084	27.6
	Households with more than two dogs	563	14.3
	Households that already had a positive dog/dogs	656	16.7
	Households with a positive dog/dogs currently	341	8.7
	Households that already had a positive dog/dogs and currently have positive dog/dogs	112	17.0
*Area of influence of household (buffer of 100m)*	*>* 0 ≤ 10 dogs	1,402	35.8
	*>* 10 ≤ 20 dogs	1,483	37.8
	*>* 20 ≤ 30 dogs	576	14.7
	*>* 30 ≤ 40 dogs	248	6.3
	*>* 40 ≤ 58 dogs	185	4.7

Diagnoses summarize the serological diagnoses and the results for investigated dogs; households represent the dog’s guardian address; the area of influence of household counts the dogs inside a buffer of 100m of radius around the household.

The maximum number of dogs per household was 17, but the mode was 1, and the mean was 1.67, with a standard deviation of 1.08. In an area of influence of a household (*a* = 31,374m2), the maximum number of dogs was 58, the mode 11, mean 16, and the standard deviation 11.37. Households that contained only one dog represent almost 60% of the domiciles, two dogs 27.6%, and more than three 14%.

Analyzing the census tracts, the odds ratio (OR) for the number of CVL and the examined dogs was 1.37 (Table 2). OR for CVL increased proportionally to the number of dogs. For households that contained only one dog was 0.40 and increased 242% for those with two dogs (OR = 1.39); and 97% when more than two dogs (OR = 2.70). For households that already had a positive dog, the OR was 2.73.

**Table 2:** Binary logistic regression of canine visceral leishmaniasis diagnostic, dog count in the buffer zone extraction versus the number of positive dogs and human cases in Bauru.

Model	Description	Odds Ratio	(95% CI)	P-value
*CVL and household*	Already had a dog/dogs with VL	2.73	2.14–3.48	0.000*
	1 dog/household	0.40	0.32–0.50	0.000*
	2 dogs/household	1.39	1.10–1.76	0.006*
	>2 dogs/household	2.70	2.09–3.48	0.000*
	Number of examined dogs	1.37	1.27–1.48	0.000*
*CVL and Area of influence of household (buffer of 100m)*	Already had a dog/dogs with VL	2.99	2.60–3.44	0.000*
	Number of examined dogs	1.10	1.09–1.11	0.000*
	≤10 dogs	0.23	0.20–0.26	0.000*
	>10 ≤ 20 dogs	1.25	1.10–1.42	0.001*
	>20 ≤ 30 dogs	2.76	2,28–3,35	0.000*
	>30 ≤ 40 dogs	7.73	5,25–11,39	0.000*
	>40 ≤ 58 dogs	7.29	4,69–11.34	0.000*
*Human cases and Area of influence of household (buffer of 100m)*	Number of positive dogs	1.16	1.09–1.24	0.000*
	Number of canine samples	1.02	1.01–1.02	0.000*
	≤10 dogs	0.75	0.64–0.88	0.000*
	>10 ≤ 20 dogs	1.16	1.00–1.35	0.053
	>20 ≤ 30 dogs	0.77	0.62–0.96	0.022*
	>30 ≤ 40 dogs	1.27	0.95–1.70	0.104
	>40 ≤ 58 dogs	2.61	1,93–3,53	0.000*

Dependent variables are CVL and HVL cases. Explanatory variables are the number of dogs, infected dogs, and the condition of the households that already had an infected dog/dogs.

*statistical significance.

Similarly, OR for the area of influence of household (buffer of 100m) also increased according to the dogs’ population. From 10 to 20 dogs, OR was 1.25 and increased 120% for 21 to 30 dogs (OR = 2.76). The influence area of households with more than 30 dogs increased by more than 150% (OR>7). In an area of influence of household, households that already had a positive dog/dogs with VL increase the chances of CVL 299% (OR = 2.99), analogous to the condition of households that already had dog/dogs with VL, in which the OR was 2.73.

Considering the human cases in an area of influence of household, the number of dogs increased the chances 102%, and the number of positive dogs 116%, demonstrating the association between canine and human VL. The number of dogs increased the chances 261% for more than 40 dogs.

### 3.5. Spatial risk

**Fig 6:**
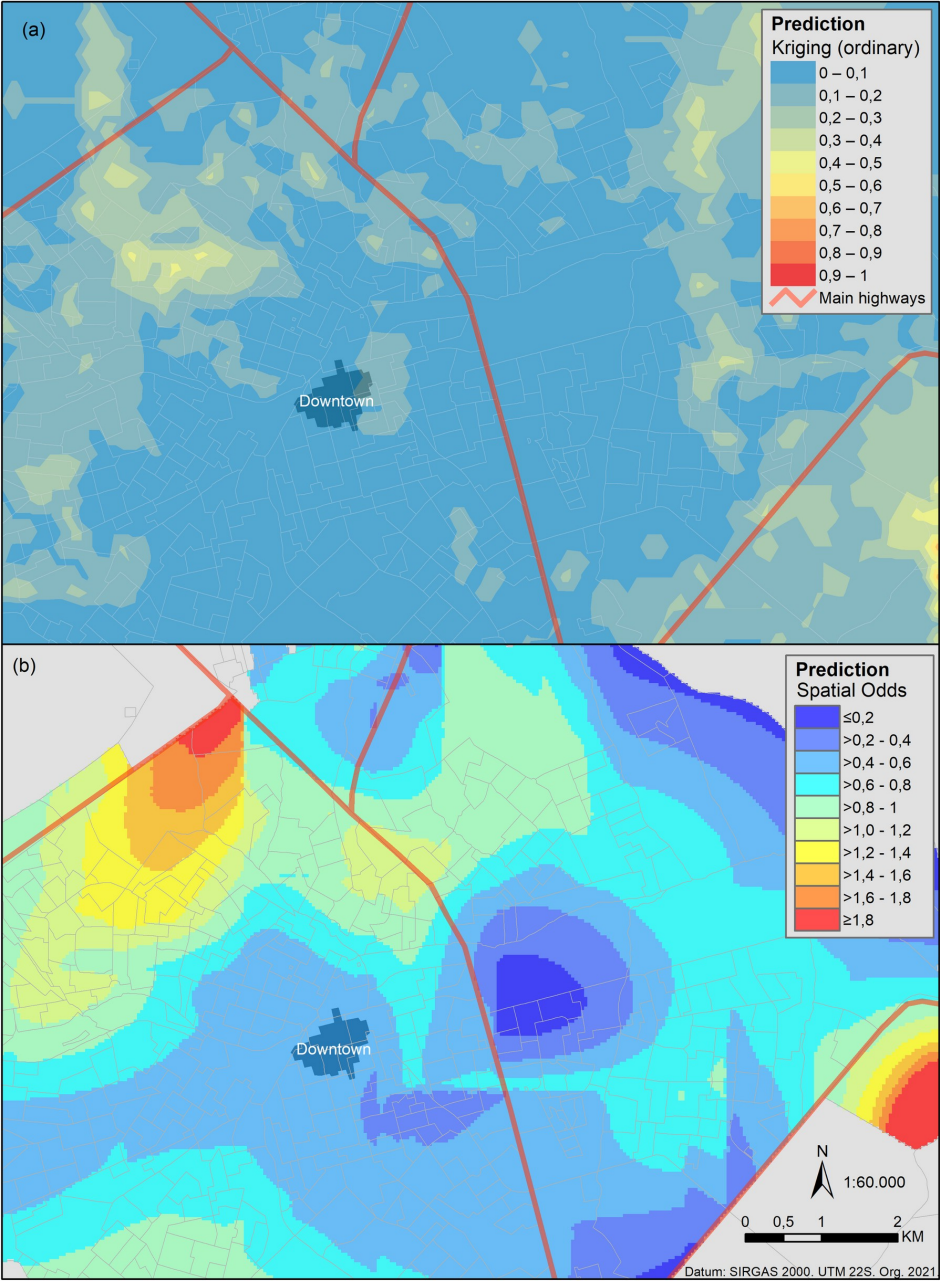
Predicting the risk for canine visceral leishmaniasis using geospatial methods. Spatial prediction models using the dog population. Risk is scaled from low (blue) to high (red), as shown by the legends. (a) Geostatistical approach using the ordinary Kriging method. (b) Generalized additive model.

Considering high OR for CVL according to the number of dogs, we created the spatial models using dogs as a predictor. Fig 6 shows that both models (geostatistical and GAM) were considerable commonality in the spatial pattern regarding the higher odds ratio areas. The higher risk is in the city’s borders, especially in the northwest and in the southeast. The last one can be a border effect. Overall, both models are spatially consistent with the Kernel density ratio map (Fig 5).

### 3.6. Cross-validation

**Fig 7:**
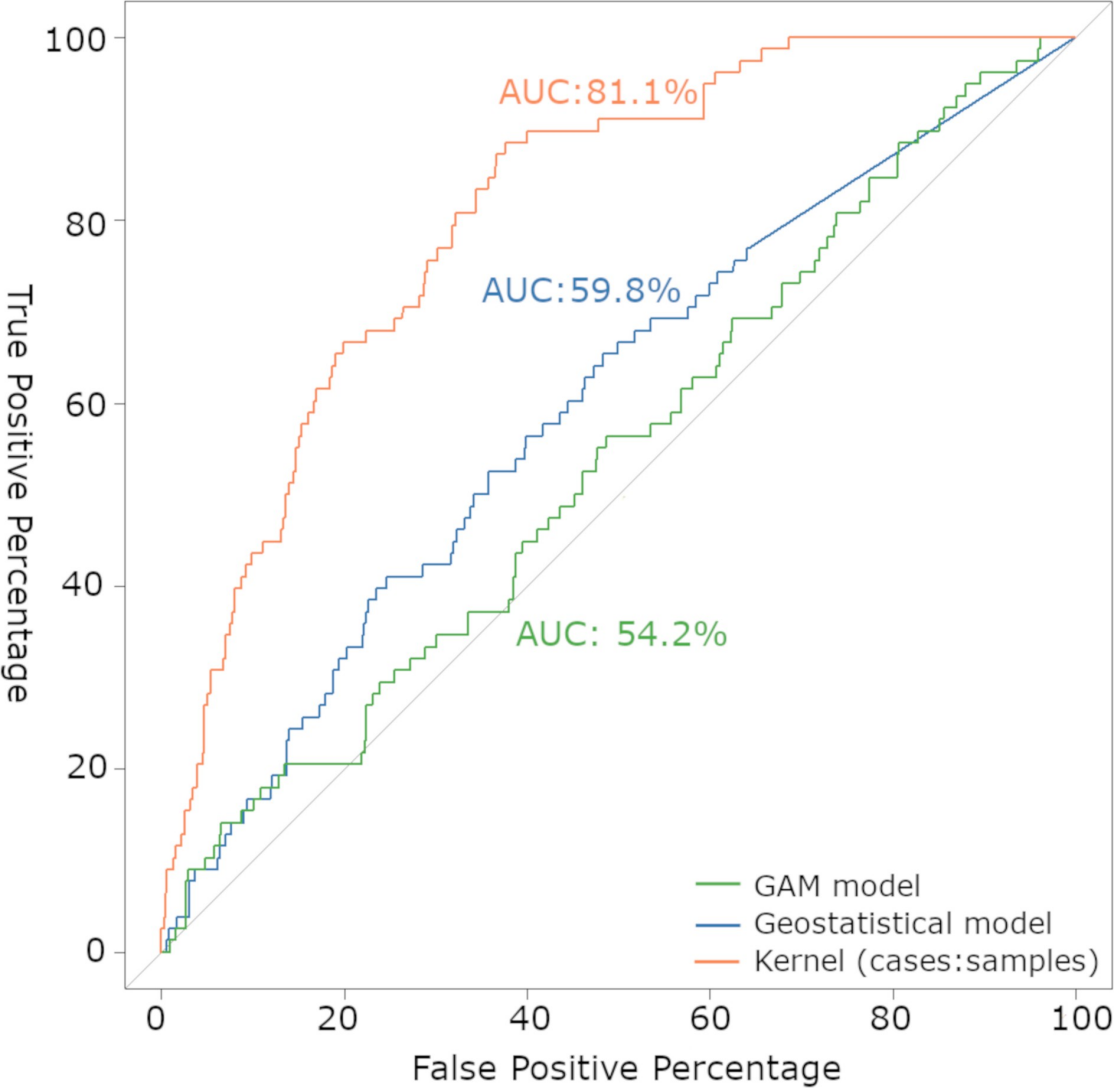
Area under the receiver operating characteristic (ROC) for canine visceral leishmaniasis models. For each model, the AUC was calculated with 95% confidence intervals. The best model in predicting canine risk disease was the Kernel density ratio map.

We plotted the Kernel density ratio, Geostatistical, and GAM models in a receiver operating characteristic (ROC) curve (Fig 7). The Kernel density ratio map presented the best threshold of 0.059, a sensitivity of 88%, a specificity of 62%, and an accuracy of 64%. The geostatistical model presented the best threshold of 0.075, a sensitivity of 65%, a specificity of 52%, and an accuracy of 53%. The GAM model presented the best threshold of 0.076, a sensitivity of 88%, a specificity of 19%, and an accuracy of 25%. The first had an AUC of 0.81 (CI 0.76–0.85), the second of 0.59 (CI 0.53–0.66) and the third of 0.54 (CI 0.47–0.60). The Kernel density ratio map presented the best performance in the ROC curve (Fig 7).

## 4. Discussion

In the current study, we found a CVL TR DPP® sero-reaction rate of 8.1% (535/6578) and 5.6% in TR DPP® confirmed by ELISA (369/6578), likely consistent with an endemic area of Sao Paulo state, Araçatuba, where the average prevalence between 2010 and 2015 was 6.8% [26]. Studies in other states of Brazil found a variable range of values of prevalence, for instance, 4.38% in Londrina, in Paraná state [27]; 8.1% in the communities of Cuiabá river [28], and 19.2% in Rondonópolis [29], both in the state of Mato Grosso; 4.16% in Belo Horizonte, in the state of Minas Gerais [30]; and a high prevalence of 50.3% in Buerarema, in the state of Bahia [31]. In Bauru, we found different spatial prevalences, ranging from 0 to 50%. Lamattina et al. (2019) found prevalences per site varying from 0 to 80% [32] and Carvalho et al. (2018) from 0 to 35% [29].

Particularly, prevalence can reveal bias once it may not represent the real number of canines. Overall, the serosurveys are directed to human case areas or areas of a suspect or identified CVL case [6, 20]. Historically, in Bauru, some neighborhoods have never performed a serosurvey before this study. On the other hand, some neighborhoods were investigated more than once since the first human case appearance, recognized by its recurrence of CVL. Our study planned the serosurvey to collect a large number of dog samples in different neighborhoods, giving a panorama of VL’s endemicity and spatial epidemiological profile in a short time. Nevertheless, sampled canines comprised less than 7% of the estimated dog population (6,578/99,815 dogs).

In the present study, our scale is the household instead of only the dogs, identifying spatial characteristics regarding the domiciles and dog population. We highlight that on the household scale, the positivity rate of domiciles that contains infected dogs (8.7%) is superior to the sampled prevalence of CVL (5.6%), which emphasizes the importance of the household environment in the disease context. Clusters of households that already had CVL can point out the areas that remain a source of infection and are unnoticed. Almost all investigated areas had these clusters. Additionally, asymptomatic dogs can be highly competent [33] and remain a source of infection without being identified. They contribute to the silent endemic areas. It can turn out into highly endemic areas or possibly a human case site.

The recent expansion of VL to new endemic areas has been attributed to the adaptation of *L*. *longipalpis* (sandflies) to naïve ecological niches. The risk of expansion of VL increases in areas identified as migratory poles of attraction. Moreover, CVL has been highlighted as the primary cause of outbreaks [34]. In these areas, canine enzootic disease precedes the appearance of human cases. In our study, human cases can increase 102% according to the dog population and 116% by the CVL, demonstrating the spatial association between canine and human VL. Other studies found that the risk increased substantially for individuals when the presence of seropositive dogs [35] or previous cases of CVL in the household [36, 37]. Furthermore, we identified a clustered pattern for both human and canine cases from approximately 500m.

As we identified, households that contain CVL and the dog population can increase the odds ratio for VL. They may influence the natural foci of *Leishmania infantum* transmission to human and animal hosts, which urges specific public policies focused on education in animal health, especially in areas target as critical. We found the same mean number of dogs per household (1.6), as reported in previous research [19]. However, about 15% of the domiciles contain more than three dogs. They should be monitored and investigated as possible infection sites. In the neighborhoods where the canine population is large, animal health assistance is required. Therefore, canine population dynamics must be considered in public policies.

Our results revealed that the risk of increasing CVL or human cases oscillated by areas. Of note, kernel maps studies have used the total number of cases or applied a constant [30]. Our study used the number of cases and samples, which gives a visualization of the risk. In accordance, a study [29] found a similar pattern of critical areas in the city’s borders. This peripheral spatial pattern seems to be expected in small and medium-sized cities of similar urbanization processes in low and middle-income countries where VL is endemic. The Kernel density ratio map was the best in the ROC curve, showing the potential of spatial analysis tools. Studies that use spatial models predicting disease risk are promising for decision-making in the control of VL.

Regarding leishmaniasis, such studies use machine learning for cutaneous leishmaniasis vectors prediction [38] or human cases prediction [39]. Nonetheless, studies using a machine learning-based approach of CVL risk prediction considering the number of dogs are still incipient. Bi et al. stress that future research about VL should focus on spatial simulation and agent-based simulation [40]. Machine learning is a novel approach that allows the forecast of disease risk. It can anticipate disease transmission dynamics and identify disease control strategies to fight endemic and emerging diseases [40]. Our models bring new insights for thinking VL through dogs from a social perspective, which has been one of the most debatable points of control programs and tends to be of low priority in the context of general public health.

It is a time of changing public policies in relation to VL. The general principles that guided the past control programs are now questionable. Guided by the VLCP, Brazilian municipalities have presented operational difficulties in executing VL control strategies [41]. Additionally, we emphasize the unavailability of the proven effectiveness of technical alternatives for laboratory diagnosis, identification, and elimination or protection of reservoirs [42].

In Brazilian cities, culling dogs has been recommended as a control measuring to reduce VL [20, 43], which creates a dog stigma. Nonetheless, culling dogs is highly controversial, considering the time between diagnosis and action. Also, the rapid replacement of euthanized dogs, entrance of new animals into the households [44–46], and the persistent disease spread challenges public policies [13, 42, 47, 48]. On the other hand, other strategies, such as the use of dog collars with insecticides for sand flies, or vaccination [49–51], have been highly encouraged due to their effectiveness in reducing the population of vector, parasitic load, and potentially the VL transmission [52–55].

Furthermore, vast territorial areas should be treated by priority order, emphasizing different profiles of VL. The decision-making should be supported by an integrated approach, considering the genetic diversity of vectors [56, 57] and the protozoa [58, 59] but integrated with education, health, and environment, including vectors, causal agents, canines, households, population density, urbanization, industries, and environmental factors, such as vegetation, water bodies, temperature, and precipitation. Animal health needs to be discussed in public policies without its stigma. Furthermore, VL should be addressed in the context of One Health [42].

To conclude, this paper had several limitations that should be recognized. Firstly, we had to use the census tract information based on the human population to calculate the dog population grid because of the lack of animal information. This could be solved with an updated canine census, hardly achieved in low and medium-income countries. Secondly, the performance of our spatial models had medium and low accuracy, although the critical areas being commonly similar to the Kernel density ratio map of higher performance. A better model’s performance could be improved with an updated census and adding real-world covariates when data become available.

Yet, there are research gaps concerning VL, and many areas of study remain unexplored. It remains the question of balancing the effectiveness and costs involved in such a VL control plan [40]. As future work, the next step of our study is to analyze the canines’ role with new insights of controlling VL, for instance, the canine cohort of insecticide-impregnated collars, vaccination, and treatment in different areas of this endemic site, as an individual and collective measure in the environment.

## 5. Conclusions

As a rule of thumb, one can say that the number of dogs and the households impact the risk for maintenance of natural foci of *Leishmania infantum* transmission to human and animal hosts in endemic areas for VL. Overall, this investigation serves as a case study for regional and global applications. It reveals the importance of canines on the household scale in low and middle-income countries. It is time for changing VL public policies using a targeted plan of priority through spatial analysis. This statement invites further investigations regarding VL characteristics involving socioeconomic and environmental variables in the context of One Health.

## Supporting information

S1 Fig Survey applied in the collection of dog’s blood. The survey was applied at the moment of the collection of dog´s blood to detect anti-*leishmania* antibodies. It is a short inquiry once the study covered a large number of dogs, and it was conducted by agents of the center for zoonoses control (not specialists). After the diagnose results, the guardians of the positive dogs were notified to schedule an appointment with a veterinarian.
(PDF)

S2 Fig K-function for visceral leishmaniases at distances. The red line is the observed values. The blue line is the expected for a random sample. Dashed lines represent the superior and inferior envelopes for statistical significance. (a) households with CVL currently; (b) human cases (2003–2019) of VL in Bauru, São Paulo, Brazil.
(PDF)

S3 Fig Kernel map for canine cases of visceral leishmaniasis. We performed a Kernel density map for the total number of canine cases using a bandwidth of 500m (approximately the minimal concentration of K-function). We select the default cells and the output in meters square.
(PDF)

S4 Fig Kernel map for canine samples. We performed a Kernel density map for the total number of dog samples using a bandwidth of 500m (approximately the minimal concentration of K-function). We select the default cells and the output in meters square.
(PDF)

S5 Fig Cluster map for households that have CVL currently. This is an example of a cluster map for the households that have CVL currently. For each category, cluster maps were created: i)households that have CVL; ii) households that already had CVL; iii) households that already had and currently have CVL. The coldspots and the non-significant data were excluded in the final cartographic representation (Fig 4).
(PDF)

S6 Fig Semivariogram for canine visceral leishmaniasis and the number of canines. The stable theoretical model was adjusted to the points according to the parameters described below.
(PDF)

S7 Fig Features of the grid of the GAM model. To calculate the number of dogs per domiciles, we used the study of Alves et al. 2005, an investigation conducted in the cities of the state of São Paulo, considering a ratio of 1:4 dogs/persons. We calculated the number of dogs based on the human population census tract (Matsumoto et al., 2021). We then used the number of households (IBGE,2010) to find the number of dogs at that point (centroid). Finally, a fishnet of 1000 cells versus 1000 cells was created to extract the point value of the number of dogs interpolation.
(PDF)

S8 Fig Interpolation of the number of dogs using Iverse Distance Weighted (IDW). This method interpolates the estimative of the cell values using the average of the points in each region. We used the census tract data and the estimative of dogs according to Alves et al. 2005. The map shows a higher number of dogs per domicile (brown to white) in the city’s outskirts and fewer dogs in the central areas (green to yellow). The grid (S7 Fig) extracted the IDW values of the correspondent location of each point. The grid can not be seen on the cartographic scale of 1:50,000, but it is visible on the scale of 1:5,000.
(PDF)

S1 File Parameters of the semivariogram. We adjusted both datasets (CVL cases and the number of dogs) using a stable model.
(PDF)

S2 File AIC results for each span. We select the best (minimal) Akaike information criterion (AIC) for choosing the span function of our GAM model. The best AIC = 1704, span = 0.15.
(PDF)

S1 Data (ZIP)

## Abbreviations

API: application programming interface
CVE: epidemiological surveillance center
CVL: canine visceral leishmanisis
ELISA: Enzyme-linked immunosorbent assay
GAM: generalized additive model
GIS: geographic information system
HVL: human visceral leishmanisis
IDW: inverse distance weighted
OR: odds ratio
ROC: receiver operating characteristic
TR DPP®: rapid test dual-path plataform Biomanguinhos
VL: visceral leishmaniasis
VLCP: Brazilian visceral leishmaniasis surveillance and control program
