# Supplementary material for: Impact of the dog population and household environment for the maintenance of natural foci of Leishmania infantum transmission to human and animal hosts in endemic areas for visceral leishmaniasis in Sao Paulo state, Brazil
Source: PLoS One. 2021 Aug 31;16(8):e0256534. doi: 10.1371/journal.pone.0256534 (PMC8407543; doi:10.1371/journal.pone.0256534)
Supplement: S1 Fig — The survey was applied at the moment of the collection of dog´s blood to detect anti-leishmania antibodies. It is a short inquiry once the study covered a large number of dogs, and it was conducted by agents of the center for zoonoses control (not specialists). After the diagnose results, the guardians of the positive dogs were notified to schedule an appointment with a veterinarian. (PDF) [file pone.0256534.s001.pdf]

Full name (guardian):			ID ____.____.____-___	Cellphone:
Full address:				
Dog's name:	ID (dog):	Breed: ☐ Poodle ☐ Yorkshire ☐ Boxer ☐ Dachshund ☐ Mixed ☐ Pinscher ☐ Shih Tzu ☐ Labrador ________ (other)	Symptoms ________ (other) ☐ sadness ☐ slimming ☐ long nails ☐ wounds ☐ flaking ☐ conjunctivitis	Obs.:
Sex: ☐ Female ☐ Male	Age: ☐ Puppy ☐ Young ☐ Adult	Fur: ☐ Black ☐ White ☐ Black-White ☐ Light Brown ☐ Dark Brown ☐ White-Brown ☐ Black-Brown ☐ White-Black-Brown		
Wearing collar (insectiside)? ☐ No ☐ Yes		Result (DPP TR) ☐ Positive ☐ Negative ☐ Indeterminate	Had a dog with VL? ☐ Yes ☐ No	Agree to participate (cohorts)? ☐ Yes ☐ No
