# Supplementary figures and images for: Impact of the dog population and household environment for the maintenance of natural foci of Leishmania infantum transmission to human and animal hosts in endemic areas for visceral leishmaniasis in Sao Paulo state, Brazil
Source: PLoS One. 2021 Aug 31;16(8):e0256534. doi: 10.1371/journal.pone.0256534 (PMC8407543; doi:10.1371/journal.pone.0256534)

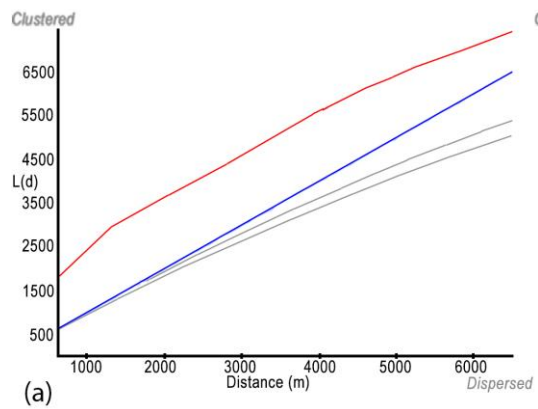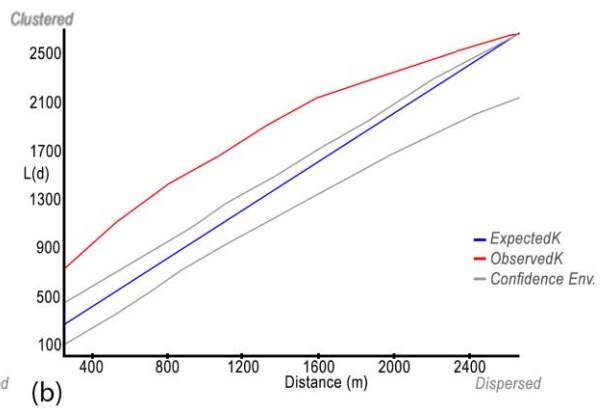

Supplement: S2 Fig — The red line is the observed values. The blue line is the expected for a random sample. Dashed lines represent the superior and inferior envelopes for statistical significance. (a) households with CVL currently; (b) human cases (2003–2019) of VL in Bauru, São Paulo, Brazil. (PDF) [file pone.0256534.s002.pdf]

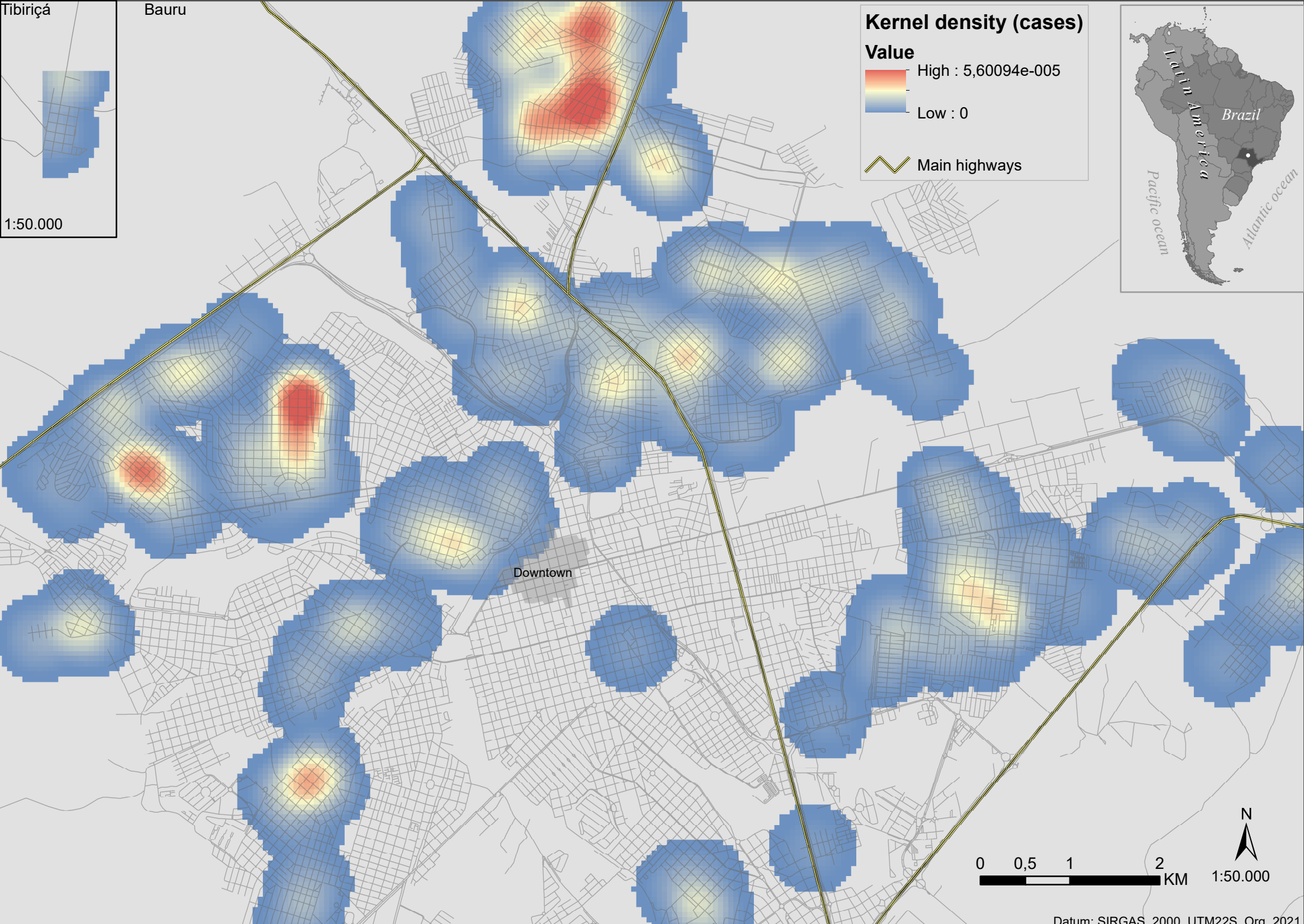

Supplement: S3 Fig — We performed a Kernel density map for the total number of canine cases using a bandwidth of 500m (approximately the minimal concentration of K-function). We select the default cells and the output in meters square. (PDF) [file pone.0256534.s003.pdf]

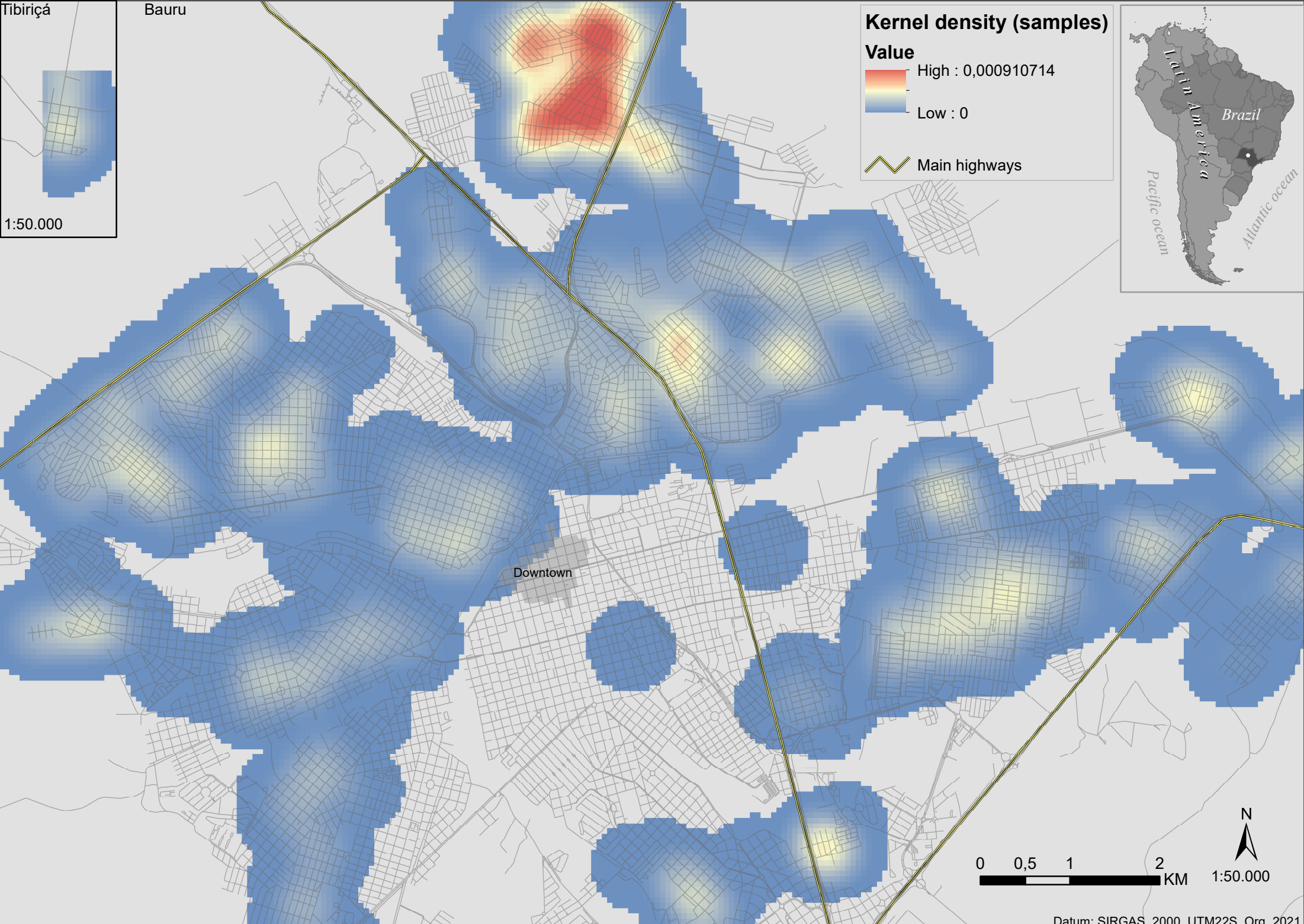

Supplement: S4 Fig — We performed a Kernel density map for the total number of dog samples using a bandwidth of 500m (approximately the minimal concentration of K-function). We select the default cells and the output in meters square. (PDF) [file pone.0256534.s004.pdf]

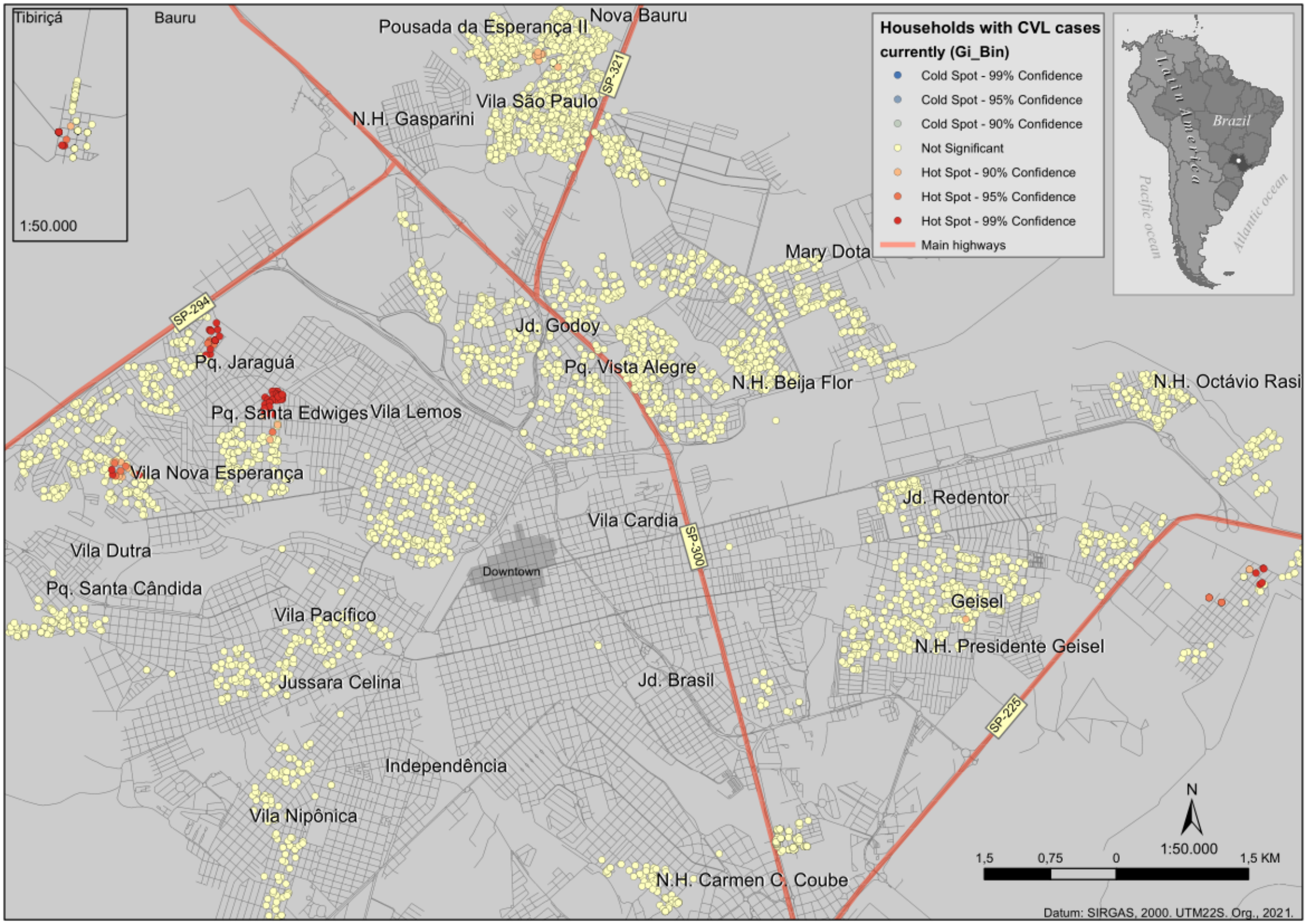

Supplement: S5 Fig — This is an example of a cluster map for the households that have CVL currently. For each category, cluster maps were created: i)households that have CVL; ii) households that already had CVL; iii) households that already had and currently have CVL. The coldspots and the non-significant data were excluded in the final cartographic representation (Fig 4). (PDF) [file pone.0256534.s005.pdf]

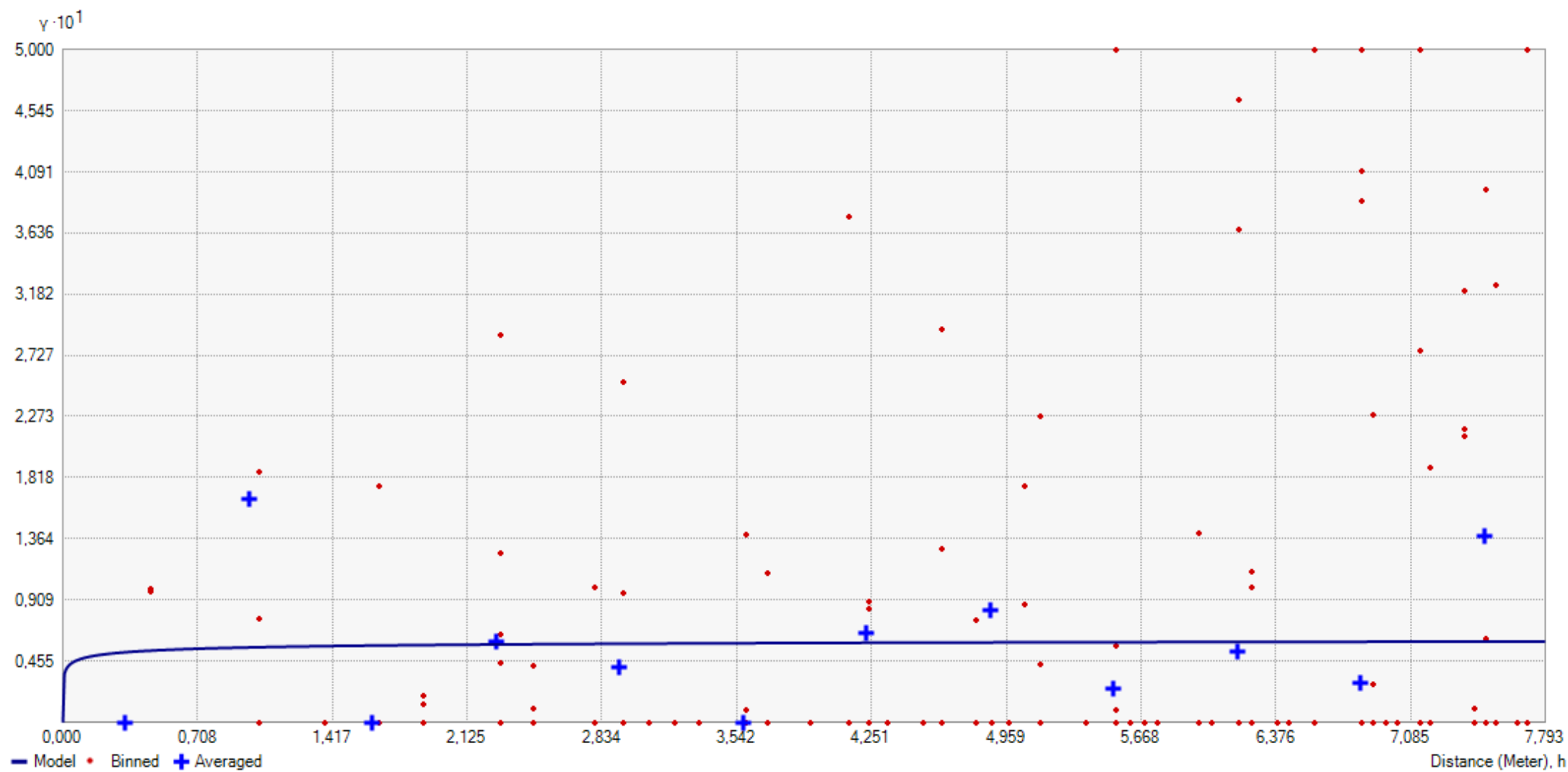

Supplement: S6 Fig — The stable theoretical model was adjusted to the points according to the parameters described below. (PDF) [file pone.0256534.s006.pdf]

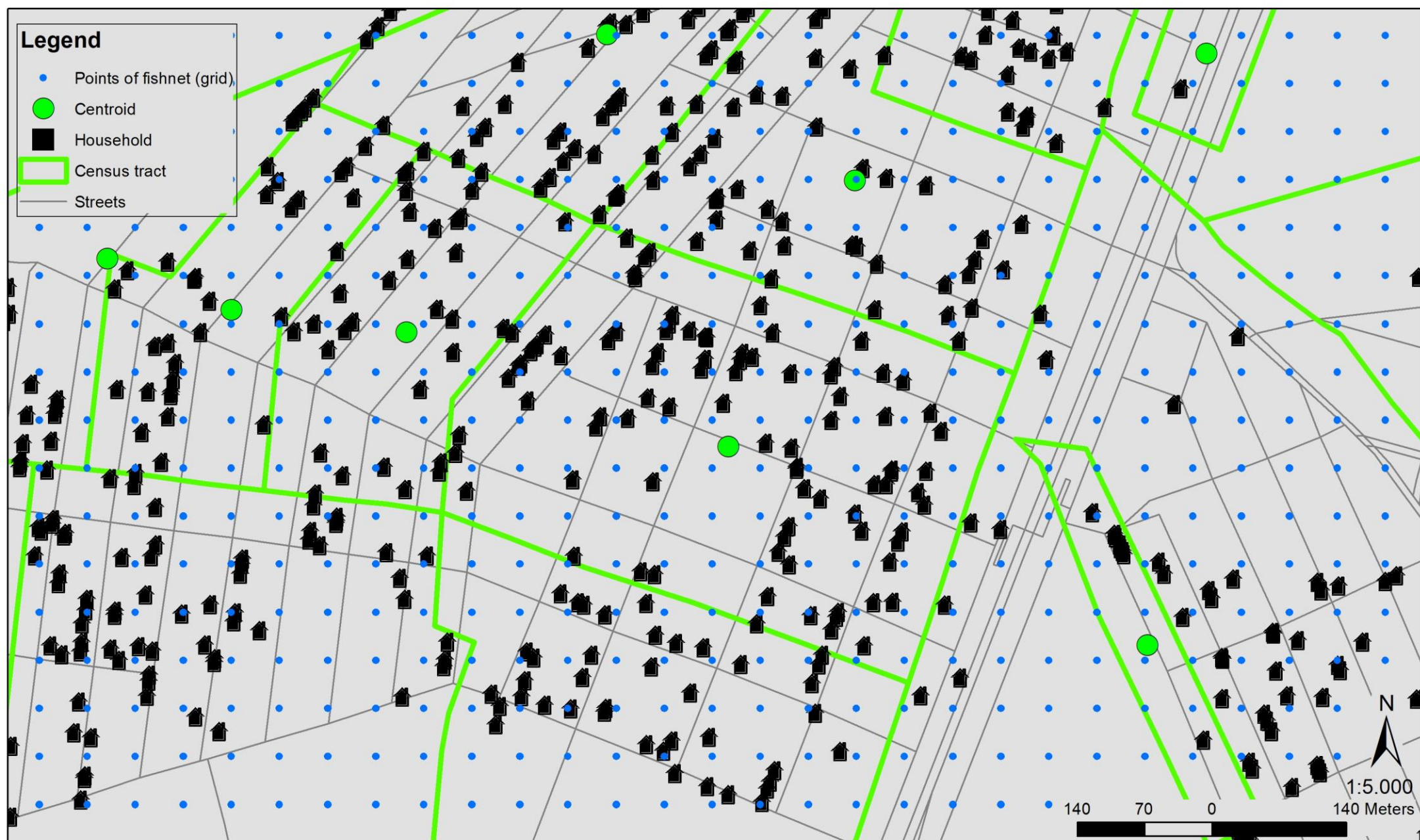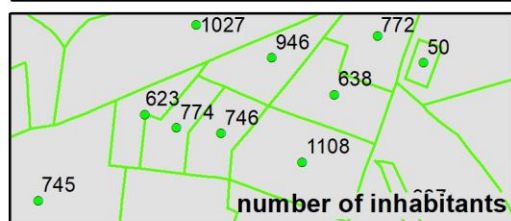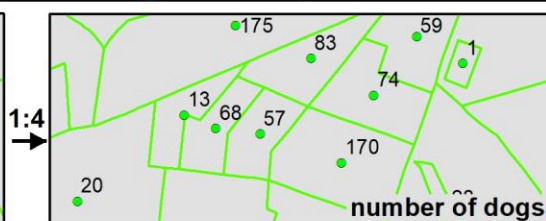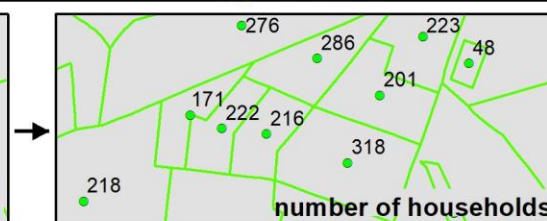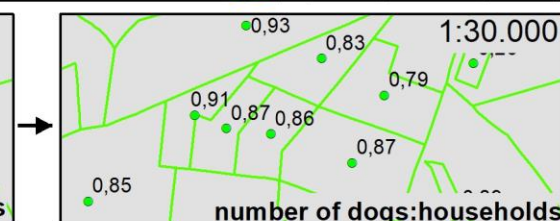

Supplement: S7 Fig — To calculate the number of dogs per domiciles, we used the study of Alves et al. 2005, an investigation conducted in the cities of the state of São Paulo, considering a ratio of 1:4 dogs/persons. We calculated the number of dogs based on the human population census tract (Matsumoto et al., 2021). We then used the number of households (IBGE,2010) to find the number of dogs at that point (centroid). Finally, a fishnet of 1000 cells versus 1000 cells was created to extract the point value of the number of dogs interpolation. (PDF) [file pone.0256534.s007.pdf]

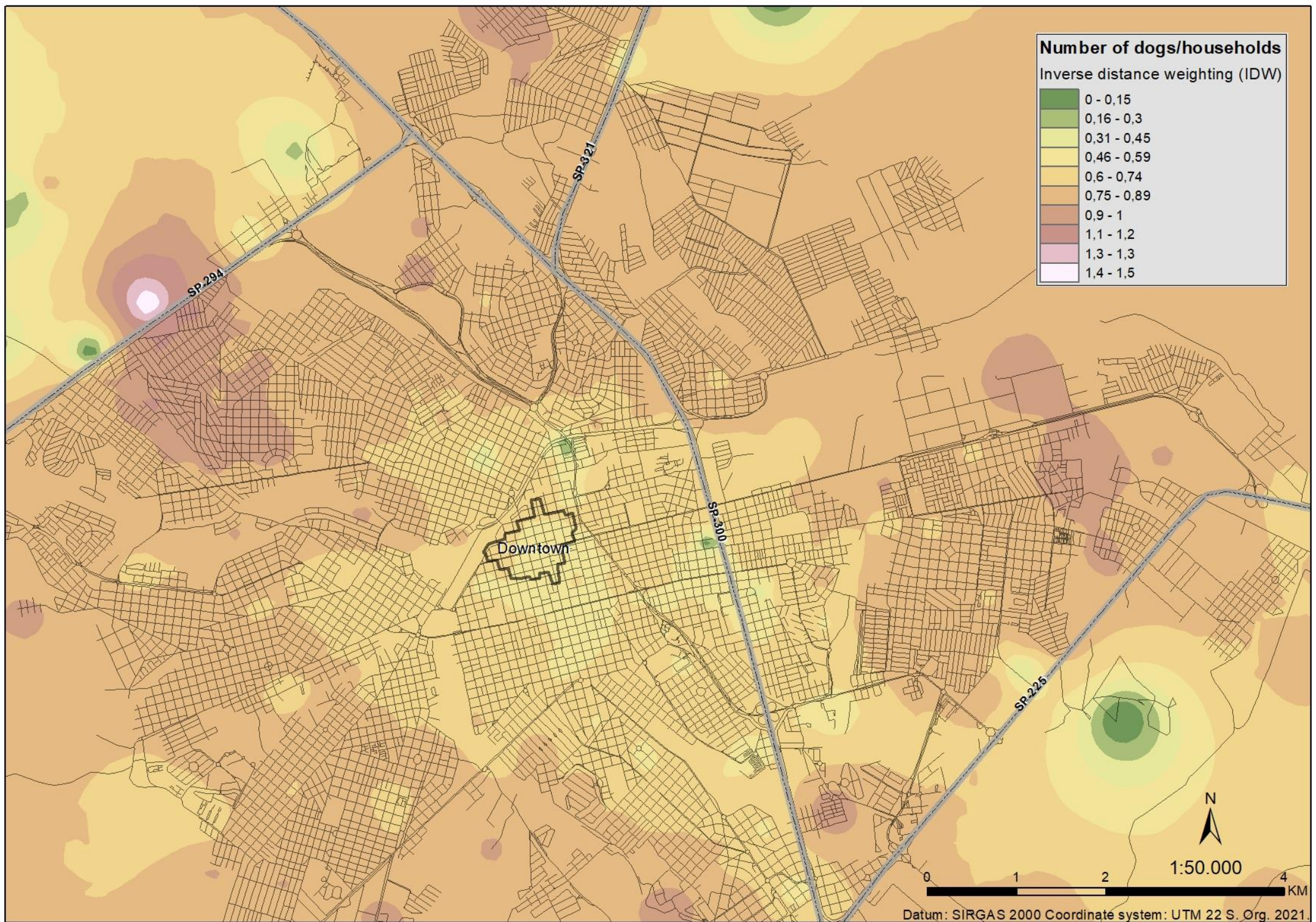

Supplement: S8 Fig — This method interpolates the estimative of the cell values using the average of the points in each region. We used the census tract data and the estimative of dogs according to Alves et al. 2005. The map shows a higher number of dogs per domicile (brown to white) in the city’s outskirts and fewer dogs in the central areas (green to yellow). The grid (S7 Fig) extracted the IDW values of the correspondent location of each point. The grid can not be seen on the cartographic scale of 1:50,000, but it is visible on the scale of 1:5,000. (PDF) [file pone.0256534.s008.pdf]
