# Supplementary material for: Impact of the dog population and household environment for the maintenance of natural foci of Leishmania infantum transmission to human and animal hosts in endemic areas for visceral leishmaniasis in Sao Paulo state, Brazil
Source: PLoS One. 2021 Aug 31;16(8):e0256534. doi: 10.1371/journal.pone.0256534 (PMC8407543; doi:10.1371/journal.pone.0256534)
Supplement: S1 File — We adjusted both datasets (CVL cases and the number of dogs) using a stable model. (PDF) [file pone.0256534.s009.pdf]

Input datasets
-**Dataset**
Type
Feature Class
Data field 1
LVC_PROJ
Records
2792

-**Dataset 2**
Type
Feature Class
Data field 1
Dogs
Records
2792

-**Method CoKriging**
Type
Ordinary
Output type
Prediction
-**Dataset #**
1
Trend type
None
-**Searching neighborhood**
Standard
Neighbors to include
5
Include at least
2
Sector type
Four and 45 degree
Major semiaxis
182.670648783572
Minor semiaxis
182.670648783572
Angle
0
-**Dataset #**
2
Trend type
None
-**Searching neighborhood**
Standard
Neighbors to include
5
Include at least
2
Sector type
Four and 45 degree
Major semiaxis
182.670648783572
Minor semiaxis
182.670648783572
Angle
0
-**Variogram**
[Semivariogram; Semivariogram]
Number of lags
12
Lag size
0.649446959339
Nugget
[0; 0.465525660772]
Measurement error %
[100; 100]
Shift
[0; 0; 0; 0]
-**Model type**
Stable
Parameter
0.2
Range
3.8974342
Anisotropy
No
Partial sill
[0.062007611094; 0.045112598095; 0.045112598095; 0.660324964631]
