# Supplementary material for: Impact of the dog population and household environment for the maintenance of natural foci of Leishmania infantum transmission to human and animal hosts in endemic areas for visceral leishmaniasis in Sao Paulo state, Brazil
Source: PLoS One. 2021 Aug 31;16(8):e0256534. doi: 10.1371/journal.pone.0256534 (PMC8407543; doi:10.1371/journal.pone.0256534)
Supplement: S2 File — We select the best (minimal) Akaike information criterion (AIC) for choosing the span function of our GAM model. The best AIC = 1704, span = 0.15. (PDF) [file pone.0256534.s010.pdf]

**Span**	**AIC training**
0.05	1749.292
0.10	1709.256
**0.15**	**1704.05**
0.20	1705.583
0.25	1709.209
0.30	1709.771
0.35	1710.728
0.40	1710.429
0.45	1710.122
0.50	1709.284
0.55	1708.946
0.60	1709.224
0.65	1707.15
0.70	1707.061
0.75	1707.243
0.80	1707.519
0.85	1707.684
0.90	1708.287
0.95	1708.906
